# Auranofin Synergizes with Cisplatin in Reducing Tumor Burden of NOTCH-Dependent Ovarian Cancer

**DOI:** 10.1158/2767-9764.CRC-25-0190

**Published:** 2025-10-10

**Authors:** Robert J. Lake, Parisa Nikeghbal, Irina V. Lagutina, Kimberly K. Leslie, Mara P. Steinkamp, Hua-Ying Fan

**Affiliations:** 1 Program in Cell and Molecular Oncology, University of New Mexico Comprehensive Cancer Center, University of New Mexico Health Science Center, Albuquerque, New Mexico.; 2 Division of Molecular Medicine, Department of Internal Medicine, University of New Mexico Health Science Center, Albuquerque, New Mexico.; 3 Biomedical Sciences Graduate Program, University of New Mexico Health Science Center, Albuquerque, New Mexico.; 4 Animal Models Shared Resource, Comprehensive Cancer Center, University of New Mexico, Albuquerque, New Mexico.; 5 Department of Pathology, University of New Mexico School of Medicine, Albuquerque, New Mexico.

## Abstract

The NOTCH pathway regulates cell proliferation, differentiation, and stem cell maintenance. Thus, aberrant NOTCH activation plays a key role in cancer initiation, progression, and chemoresistance. Mutations and amplification of NOTCH pathway genes have been identified in high-grade serous ovarian cancers and are associated with poor clinical outcomes. Among the four NOTCH receptors, NOTCH3 alterations were strongly correlated with poor overall survival. Previously, we identified auranofin, an oral gold salt therapeutic compound, as a novel NOTCH pathway inhibitor that disrupts the DNA binding of RBPJ, the major downstream transcriptional effector of the NOTCH pathway. In this study, we surveyed the response of eight ovarian cancer cell lines to auranofin and found IC_50_ values ranging from 1.7 to 12 μmol/L, with NOTCH3-negative SKOV3 cells having the highest IC_50_ value. In NOTCH-dependent OVCAR3 cells, auranofin synergized with cisplatin to enhance cell death. Importantly, auranofin treatment led to a dose-dependent decrease in RBPJ occupancy at the NOTCH-dependent promoters, *HES1* and *HES4*. Furthermore, knocking down *NOTCH3* in OVCAR3 cells significantly decreased sensitivity to auranofin, further supporting the notion that NOTCH3 signaling is a major target of auranofin. Moreover, auranofin increased cisplatin efficacy in an OVCAR3-derived xenograft mouse model. Using eight patient-derived cancer organoid models, we found that auranofin increased cisplatin efficacy in killing cancer organoids generated from clinically platinum-sensitive patients but also restored platinum response in a subset of organoid models developed from platinum-resistant patients. These studies underscore the potential of auranofin to improve platinum-based cancer therapy, particularly in NOTCH3-expressing cancers.

**Significance:**

NOTCH signaling underlies cancer initiation, progression, and chemoresistance. Our study revealed the potential of auranofin as a NOTCH pathway inhibitor to enhance the efficacy of platinum-based ovarian cancer therapy.

## Introduction

Ovarian cancer is all too often a deadly disease, primarily due to late-stage diagnosis, when the cancer has already spread within the peritoneal cavity. First-line therapy consists of cytoreductive surgery, followed by adjuvant platinum/taxane chemotherapy. Although this standard treatment yields a response rate of more than 80% in advanced ovarian cancer, the median progression-free survival is only 18 months. Some patients with relapse can be treated a second time with platinum/taxane, but chemoresistance is a major obstacle. Multiple second-line therapies are available, but none are effective (1, 2). Accordingly, new options for the treatment of both primary and relapsed ovarian cancers are urgently needed.

The NOTCH signaling pathway regulates cell proliferation, survival, differentiation, and stem cell maintenance. Given the fundamental functions of NOTCH, it is not surprising that aberrant NOTCH activation has been shown to play key roles in cancer initiation, progression, and chemoresistance (3–10). A study conducted by The Cancer Genome Atlas revealed that mutations and amplifications of genes in the NOTCH pathway occur in 22% of high-grade serous ovarian cancers (HGSOC), and genetic alterations in NOTCH pathway genes are associated with poor clinical outcomes (11–13). Of the four NOTCH receptors, NOTCH3 alterations have been demonstrated to be more prevalent. NOTCH3 overexpression has been shown to lead to ovarian cancer stem cell expansion and increased resistance to platinum compounds (14). Moreover, NOTCH3 has been shown to promote anoikis resistance in ovarian cancer cell models, which is a prerequisite for the survival of cells that detach from their surroundings (14–16). Conversely, reducing NOTCH3 levels in ovarian cancer cell lines can decrease proliferation and induce apoptosis (12, 14). Altogether, a large body of data have revealed that the Notch pathway is a critical and relevant target for ovarian cancer therapy, with the Notch3 receptor being particularly important (16–20).

Unlike most cell signaling mechanisms, the NOTCH pathway is linear as it does not have an enzymatic amplification step (21–23). Therefore, this pathway is very sensitive to changes in ligand and receptor dosages caused by gene amplification, which is frequently observed in ovarian cancer. The four NOTCH receptors and five NOTCH ligands are single-pass transmembrane proteins. Pathway activation occurs through juxtacrine signaling and relies on direct cell–cell contact. Once activated, a series of proteolytic cleavages release the NOTCH intracellular domain (NICD) from the plasma membrane. The NICD then translocates to the nucleus, in which it forms a ternary complex consisting of the sequence-specific transcriptional effector RBPJ (also known as CSL) and a mastermind-like coactivator to form a core trimeric transcription activation complex (24).

To date, three main strategies have been used to develop NOTCH pathway inhibitors for cancer therapy (25). Humanized monoclonal antibodies target a NOTCH receptor or ligand to prevent receptor activation (20); however, owing to their long half-lives, the use of blocking antibodies leads to severe on-target toxicities. Another strategy is to use γ-secretase inhibitors (GSI), as γ-secretase is necessary for the release of active NICD from the plasma membrane. Unfortunately, a major limitation of this approach is that there are more than 90 known substrates of this enzyme complex; therefore, GSIs lead to off-target effects (26). A third strategy is to prevent the formation of the ternary transcriptional activation complex. The clinical value of two compounds (SAHM1 and IMF-1) has yet to be investigated (27, 28). Another compound, CB103, inhibits tumor growth, reduces tumor burden in cell line–derived xenograft (CDX) models and resensitizes tumors to oxaliplatin in patient-derived xenotransplantation experiments (29). A phase I/IIA study was conducted to examine the effect of CB103 in patients with locally advanced or metastatic solid tumors and hematologic malignancies associated with *NOTCH* alterations (NCT03422679) although ovarian cancer was not included in this study. Most importantly, in these clinical trials, few on- and off-target toxicities were associated with CB103, in contrast to NOTCH antibodies and GSIs, indicating that blocking NOTCH signals at the transcriptional level is a promising approach.

Given that RBPJ is the major transcriptional effector of NOTCH signaling, we hypothesized that inhibiting RBPJ–DNA binding would broadly downregulate aberrant NOTCH activation resulting from the overexpression or gain-of-function mutation of any upstream NOTCH pathway component (30). To identify disrupters of RBPJ–DNA interactions, we developed a flow cytometry–based assay to directly monitor RBPJ–DNA interactions using purified components. We found that the FDA-approved drug auranofin, a gold-containing compound, disrupted RBPJ–DNA interactions in a dose-dependent manner. Using a collection of cell-based assays, we further demonstrated that this novel Notch pathway inhibitor disrupted RBPJ–DNA binding and downregulated NOTCH-dependent transcriptional activation (30).

In this study, we tested the effects of auranofin on a panel of ovarian cancer cell lines, a CDX mouse model, and eight patient-derived cancer organoid (PDO) models. We demonstrated that auranofin enhanced cisplatin efficacy in NOTCH-dependent OVCAR3 cells (12, 14) in both monolayer and CDX models. We observed synergy in NOTCH3-expressing organoids generated from patients with platinum sensitivity. Strikingly, we also found that auranofin restored platinum sensitivity in two of five cancer organoids developed from platinum-resistant patients. This study underscores the potential of using auranofin to enhance platinum-based ovarian therapy and improve personalized medicine.

## Materials and Methods

### Cell culture

All ovarian cancer cell lines used in this study were of female origin and authenticated by short tandem repeat profiling. All lines for this work were provided by Dr. Leslie; Dr. Swisher (University of Washington) generously shared several of them. Specifically, OVCAR3 (RRID:CVCL_0465) was obtained from the ATCC and used for no more than 20 passages (∼8–10 weeks) after thawing. PEO4 (RRID:CVCL_2690) was purchased from Sigma and similarly limited to ≤20 passages. PEO1 (RRID: CVCL_2686), COV362 (RRID: CVCL_2420), Caov-3 (RRID: CVCL_0201), ES2 (RRID: CVCL_3509), and SKOV3 (RRID: CVCL_0532) were provided by Dr. Leslie (original stocks kindly shared by Dr. Swisher) and maintained for no more than 20 passages (∼8–10 weeks). All lines were maintained in DMEM supplemented with 10% FBS, 100 U/mL penicillin–streptomycin, and 2 mmol/L L-glutamine at 37°C in a humidified 5% CO_2_ atmosphere. Short tandem repeat authentication was performed by Bio-Synthesis, Inc. using the Whatman FTA Sample Collection Kit for Human Cell Authentication Service (cat. # 135-XV). Mycoplasma testing was conducted annually with the InvivoGen MycoStrip Mycoplasma Detection Kit, most recently in July 2024.

### Protein quantification and Western blotting

To prepare whole cell lysates, cells were collected in 1× SDS sample buffer [125 mmol/L Tris-base (pH 6.8), 2.5% SDS, and 20% glycerol] without dithiothreitol or bromophenol blue. Lysates were sonicated using a Branson 101-135-126 sonifier at 25% amplitude for 60 seconds. BCA protein assays (Thermo Fisher Scientific, # 23225) were used to quantify protein concentrations according to the manufacturer’s instructions. Dithiothreitol and bromophenol blue were then added to the cell lysates. Ten to twenty micrograms of proteins per lane were resolved on a 4% to 12% Bis-Tris SurePAGE gel in MOPS SDS buffer (GenScript, # M00653). Immunoblots were developed using either SuperSignal West Pico (#34083; Thermo Fisher Scientific) or WesternBright Quantum (Advansta, # K-12042-D10) horseradish peroxidase (HRP) chemiluminescent substrate and imaged using a Konica Processor SRX-101A.

### Antibodies

Rabbit polyclonal anti-NOTCH3 antibody was purchased from ABclonal Technology (A13522, RRID:AB_2760384, 1:2,000). HRP-conjugated goat anti-mouse IgG + IgM antibodies were purchased from The Jackson Laboratory (#115-035-044, RRID:AB_2338503, 1:10,000); antibodies against histone H3 (#9715, RRID:AB_331563, 1:2,000) and HRP-conjugated goat anti-rabbit IgG (#7074, RRID:AB_2099233, 1:3,000) were purchased from Cell Signaling Technology. Anti-actin antibody was obtained from Santa Cruz Biotechnology (SC-8432, RRID:AB_626630, 1:100). Rabbit anti-RBPJ antibody (FC32) was used at 1:200 (31).

### Chromatin immunoprecipitation

Chromatin immunoprecipitation (ChIP) was performed as described previously (30, 31). Briefly, approximately 10 million cells were fixed with 1% formaldehyde for 10 minutes and sonicated on ice at 40% amplitude (30 seconds on and 90 seconds off for 24 minutes) using a Branson 101-135-126 Sonifier. ChIP was performed using a polyclonal anti-RBPJ antibody (FC31; 1:100) and 5 μL of blocked protein-A agarose beads (Thermo Fisher Scientific, cat. # 20333). Ten micrograms of rabbit IgG was used in place of the anti-RBPJ antibody as a background control. The samples were reverse cross-linked for 16 hours at 65°C. ChIPed DNA was purified and analyzed by real-time PCR using PerfeCTa SYBR Green FastMix, Low ROX (Quantabio) in a 384-well format with a QuantStudio 5 real-time PCR system (Applied Biosystems). The enrichment of RBPJ in specific genomic regions was normalized to the total DNA input of the respective regions (2^Ct-Ctinput^). The results are expressed as fold enrichment relative to the IgG controls. The primers used are listed in Supplementary Table S1.

### Short hairpin RNA–mediated gene knockdown

Short hairpin RNAs (shRNA) targeting *NOTCH3* (GeneCopoeia, cat. # HSH011876-LVRU6GP) and a scrambled control (cat. # CSHCTR001-LVRU6GP) were used for gene silencing. Lentivirus was produced using third-generation packaging plasmids, as previously described (32). OVCAR3 and SKOV3 cells were plated at approximately 20% confluency at the time of infection. Fresh medium was added 24 hours after infection, and cells were harvested for analysis 72 hours later for Western blot analysis to monitor NOTCH3 protein levels. For stable knockdown, cells were selected with puromycin at 1 μg/mL for OVCAR3 and 2 μg/mL for SKOV3.

### Drug treatment and cell viability measurement

For established cell line drug treatments, 25,000 cells were seeded in a 96-well plate (#333-8091-W1lE1, Caplugs, Inc.) in the presence of varying concentrations of cisplatin (stock solution: 10 mmol/L in dimethylformamide, HY-17394, MedChemExpress) and auranofin (stock solution: 50 mg/mL in DMSO, Cayman Chemical). After 72 hours of treatment, cell viability was measured using the ATP-Glo Bioluminometric Cell Viability Assay (cat. #30020, Biotium), according to the manufacturer’s specifications. The signals were read using a BioTek Synergy Neo2 plate reader.

### Determination of drug interaction between cisplatin and auranofin in OVCAR3 cells

OVCAR3 cells were treated with auranofin alone (1.2, 1.8, 2.4, 3, and 6 μmol/L), cisplatin alone (4, 6, 8, 10, and 20 μmol/L), and in combination. Combination index (CI) values at effect levels ED25, ED50, ED75, and ED90 were calculated using the median-effect equation of Chou–Talalay (33) with GraphPad Prism and Microsoft Excel software. A CI < 1 indicates synergy, CI = 1 indicates additivity, and CI > 1 indicates antagonism.

### OVCAR3 xenograft model of disseminated ovarian cancer

All mouse procedures were approved by the University of New Mexico Animal Care and Use Committee (protocol no. 22-201335-HSC), in accordance with NIH guidelines for the care and use of experimental animals. For *in vivo* testing of auranofin synergy with cisplatin, the platinum-sensitive OVCAR3 cell line transduced with the bioluminescent reporter R-luciferase (PerkinElmer) was engrafted into the peritoneal cavity of NOD/SCID gamma (NSG) immunocompromised mice (RRID: IMSR_JAX:005557). Two weeks after engraftment (week 3), mice were imaged using the *in vivo* imaging system to obtain a pretreatment baseline measurement of tumor burden. Groups of five mice each were treated with auranofin, low-dose cisplatin, or their combination, as detailed in the figures. The tumor burden was assessed weekly. Mice were sacrificed if their body weight dropped by 20% from their initial body weight or if they showed abdominal distension. The percentage change in tumor burden from baseline after treatment was plotted for each mouse.

### Isolation of cancer cells from patient samples

Malignant ascites fluid was collected from consenting patients with ovarian cancer undergoing cytoreductive surgery at the University of New Mexico Comprehensive Cancer Center as detailed in Steinkamp and colleagues (34). The acquisition of patient samples was approved by the University of New Mexico Health Sciences Center Institutional Review Board (protocol no. INST1509), and the studies were conducted in accordance with the U.S. Common Rule. Eligible participants for this study must meet all inclusion and exclusion criteria, as waivers are not permitted, and study treatment cannot commence until registration. Inclusion criteria require female participants of any ethnicity, aged 18 or older, undergoing cytoreductive surgery for suspected ovarian cancer. They must provide informed consent and have a pathologic or clinical suspicion of ovarian cancer scheduled for tumor resection. Secondary inclusion criteria include a confirmed diagnosis of epithelial adenocarcinoma of the ovary, fallopian tube, or primary peritoneal cancer and a negative serum pregnancy test within 2 weeks prior to study entry for women of childbearing potential. Exclusion criteria include individuals unable to provide informed consent, minors below 18, pregnant women, and those whose final pathology does not confirm invasive epithelial ovarian, tubal, or primary peritoneal cancer. Red blood cells were lysed using an ammonium chloride solution (STEMCELL Technologies, 07800). Cancer spheroids were isolated from ascites fluid, cryopreserved in freezing media (5% DMSO/95% FBS; Gibco, Thermo Fisher Scientific), and stored at −80°C prior to processing for organoid development.

### Patient-derived xenografts

Fresh or cryopreserved spheroids isolated from the ascites fluid of patients with ovarian cancer were injected into the peritoneal cavity of in-house bred NSG mice to establish orthotopic patient-derived xenograft (PDX) models of disseminated ovarian cancer, as detailed by Steinkamp and colleagues (34). PDX-engrafted mice were euthanized at a humane endpoint when ascites fluid accumulation caused abdominal distention or when the mice showed signs of wasting. During necropsy, solid tumors and ascites fluid were collected and cryopreserved in 5% DMSO/95% FBS. PDX cancer organoids (PDXO) were generated from cryopreserved solid PDX tumors, as described below.

### Organoid formation

Development of PDOs or PDXOs followed protocols as described previously (35). Briefly, solid tumors were mechanically dissociated and incubated for 30 minutes at 37°C with rotation in digestion buffer [human wash buffer (DMEM:Ham’s F-12; Gibco, 21041025), 10 mmol/L HEPES (Gibco, 15-630-080), 1× GlutaMAX (Gibco, 35-050-061), 100 μg/mL Primocin (Invivogen, ant-pm-1), and 0.1% BSA (Sigma, A8531)] supplemented with 2 U/mL dispase II (Sigma, D4693), 1 mg/mL collagenase P (Millipore, 11213857001), and 2 μL/mL DNase I (New England Biolabs, M0303S). The digested tissue was strained through a 40 μm strainer to obtain a single-cell suspension, washed twice with human wash buffer, and centrifuged at 500 × *g* for 10 minutes after each wash. Primary ascites-derived cancer spheroids were centrifuged at 500 × *g* and resuspended in the digestion buffer.

To establish cancer organoid cultures, 50,000 cancer cells/well were embedded in 100% UltiMatrix (Cultrex, BME001-10) in 50 μL of domes in a 24-well plate. Domes were overlaid with 450 μL of organoid media [advanced DMEM:Ham’s F-12 (Gibco, 12634028)] supplemented with 1× GlutaMAX, 10 mmol/L HEPES, penicillin–streptomycin, 100 ng/mL recombinant human noggin (PeproTech, 120-10C), 250 ng/mL R-spondin-1 (R&D Systems, 4645-RS-025), 100 ng/mL FGF10 (PeproTech, 100-26), 37.5 ng/mL heregulin beta-1 (United States Biological, H2030-50), 50 ng/mL EGF (R&D Systems, 236-EG), B27 supplement (1:50, Thermo Fisher Scientific, 17504044), 1.25 mmol/L N-acetylcysteine (Sigma, A9165), 100 nmol/L ß-estradiol (Sigma, E2758), 2% Primocin, 5 mmol/L nicotinamide (Sigma, N0636), 5 μmol/L A83-01 (Tocris, 2939), 10 μmol/L Y-27632 (Sigma, Y0503),10 μmol/L forskolin (Tocris, 1099), and 250 μg/mL hydrocortisone (Sigma, H0888). The initial organoid formation from single cells took 6 to 14 days.

### PDO viability assays

The response to auranofin and cisplatin was tested in the 3D PDOs and PDXOs. These assays were performed in 96-well white plates with µClear bottoms (CELLSTAR, Sigma, M1062-40EA). Twenty to thirty organoids were seeded per well in a 1:1 ratio of UltiMatrix/organoid media as 5 μL domes, following a previously described protocol (35). The solidified domes were topped with 200 μL of prewarmed organoid medium per well. The next day, organoids were treated with cisplatin (1 or 4 μmol/L), auranofin (2.5, 5, or 7.5 μmol/L), or combinations of the two drugs as specified in the figures. DMSO (0.1%) diluted in organoid medium served as the vehicle control. After 72 hours of treatment, organoid cell viability was determined using the CellTiter-Glo 3D Cell Viability Assay (Promega, G9682), following the manufacturer’s protocol. Briefly, equal amounts of CellTiter-Glo 3D reagent were added to the medium in each well. The plates were agitated vigorously in a microplate reader for 5 minutes and left to sit at room temperature for 25 minutes to stabilize the luminescence signal. Luminescence was measured using a BioTek Synergy Neo2 multimode high-sensitivity plate reader from the University of New Mexico Comprehensive Cancer Center Flow Cytometry Shared Resource. Each test condition was repeated in five or more wells as a technical replicate. Two independent drug response studies were performed using PDO/PDXO as the biological replicates.

### Statistical analyses

GraphPad Prism (RRID:SCR_002798, version 10.4.1) and Microsoft Excel (Microsoft Excel, RRID:SCR_016137, version 16.94) were used for statistical analyses and graphical representation, with data presented as means ± SD or SEM. One-way ANOVA with Holm–Šídák multiple comparisons, unpaired *t* tests, and one- or two-tailed *t* tests were used to determine statistical significance, as specified in the figure legends.

## Results

### Ovarian cancer cells with activated Notch3 are more sensitive to auranofin

**Figure 1.**
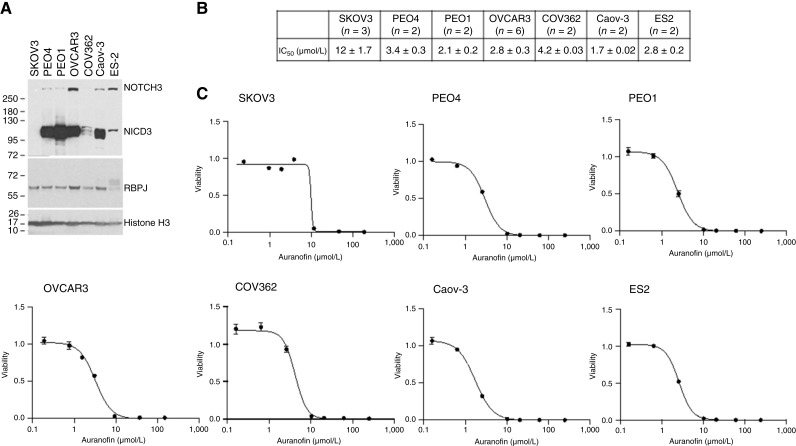
Ovarian cancer cells with activated Notch3 are more sensitive to auranofin. **A,** Western blot analysis of total cell lysates showing Notch3 and RBPJ levels in seven established ovarian cancer cell lines. Histone H3 was used as a loading control. **B,** Summary of IC_50_ values for auranofin in the seven ovarian cancer cell lines tested, shown as means ± SEM. **C,** Representative examples of auranofin dose-response curves in the ovarian cancer cell lines tested. Cells were treated with varying concentrations of auranofin for 72 hours. Cell viability was determined using the ATP-Glo Bioluminometric Cell Viability Assay. IC_50_ values were determined by fitting the data points using the four-parameter sigmoidal model.

As a first step in assessing the utility of auranofin for ovarian cancer therapy, we determined Notch3 and RBPJ protein levels in a collection of well-characterized ovarian cancer cell lines (Fig. 1A). Although all ovarian cancer cell lines contained similar levels of RBPJ protein, they had different levels of cleaved NOTCH3 (NICD3). SKOV3 cells had very little or no NICD3; COV362 and ES2 cells had low but detectable levels of NICD3; and PEO4, PEO1, OVCAR3, and CaoV3 cells had high levels of NICD3.

Next, we determined the concentration of auranofin required to inhibit 50% of the proliferative potential of the ovarian cancer cell lines (IC_50_). All seven cell lines responded to auranofin, with an approximately 10-fold difference in IC_50_ values. NICD3-low SKOV3 and COV362 cells had the highest IC_50_ values (Fig. 1B and C; refs. 12, 14).

### NOTCH3 is a key mediator of auranofin’s cytotoxicity in ovarian cancer cells

**Figure 2.**
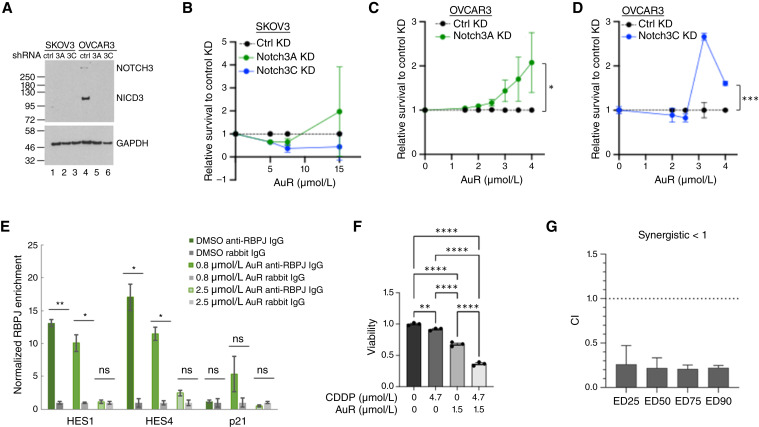
Auranofin inhibits NOTCH3 signaling in OVCAR3 cells and enhances cisplatin efficacy. **A,** Western blot showing reduced NOTCH3 protein levels following a 72-hour treatment with two different shRNAs targeting *NOTCH3* in OVCAR3 and SKOV3 cells. **B,** Auranofin response in SKOV3 cells stably expressing *NOTCH3* shRNA. Data are means ± SEM (*n* = 2 biological replicates). **C,** Auranofin response in OVCAR3 cells stably expressing *NOTCH3* shRNA_3A. Data are means ± SEM (*n* = 2 biological replicates). **D,** Auranofin response in OVCAR3 cells transiently transfected with shRNA_3C. Data are means ± SEM (*n* = 6 technical replicates). Two-way ANOVA was used for statistical analysis of B–D. *, *P* < 0.05; ***, *P* < 0.001. **E,** Representative anti-RBPJ ChIP-qPCR results in OVCAR3 cells treated with DMSO or 0.8 or 2.5 μmol/L auranofin for 24 hours. Data are means ± SEM (*n* = 3 technical replicates). RBPJ occupancy at *HES1* and *HES4* promoters decreased in a dose-dependent manner. *CDKN1A* (p21) served as a negative control. Unpaired two-tailed *t* tests were used to determine statistical significance relative to IgG. *, *P* < 0.05; **, *P* < 0.01; ns, not significant. **F,** Interaction between auranofin and cisplatin was analyzed by one-way ANOVA with Holm–Šídák multiple comparisons test (single pooled variance). **, *P* < 0.01; ***, *P* < 0.001; ****, *P* < 0.0001; ns, not significant. **G,** CI values, calculated using the Chou–Talalay median-effect method, are plotted against the fraction of cells affected (Fa). CI <1 indicates synergy. Data are means ± SD (*n* = 2 biological replicates). ED25, ED50, ED75, and ED100 = 25%, 50%, 75%, and 100% inhibition of viability, respectively. AuR, auranofin; CDDP, cisplatin; KD, knockdown.

To determine whether NOTCH3 is a primary mediator of auranofin’s cytotoxicity in ovarian cancer cells, we used shRNA targeting *NOTCH3* and examined the effect on auranofin sensitivity. We envision three scenarios. First, if NOTCH3 is not a major target of auranofin, then knocking down *NOTCH3* should have little or no impact on auranofin sensitivity. Second, if auranofin decreases cell viability primarily through the inhibition of Notch signaling, then *NOTCH3* knockdown should reduce sensitivity to auranofin. Third, if auranofin has two significant targets and NOTCH3 is one of them, then sensitivity in *NOTCH3* knockdown cells would be reduced compared with control knockdown but not abolished. We depleted NOTCH3 in SKOV3 (low NICD3) and OVCAR3 (high NICD3) cells using two independent shRNAs (*NOTCH3*A and *NOTCH3*C) alongside a matched nontargeting control (Fig. 2A). SKOV3 cells expressing either *NOTCH3*A or *NOTCH3*C shRNA were under puromycin selection for 5 days before assessment of auranofin sensitivity (Fig. 2B). OVCAR3 cells expressing *NOTCH3*A shRNA were also assayed after 5 days of puromycin selection, whereas OVCAR3 cells expressing *NOTCH3*C shRNA were assayed 48 to 72 hours after infection without selection as extended culture was not feasible due to a severe proliferation defect (Supplementary Fig. S1A and S1B). Of note, *NOTCH3*A shRNA-expressing OVCAR3 cells also displayed a similar defect, as their proliferation was severely compromised roughly 2 weeks after infection. These proliferation defects are consistent with the notion that NOTCH3 is critical for OVCAR3 cell survival.

In SKOV3 cells, NOTCH3 depletion modestly increased auranofin sensitivity at 5 and 7.5 μmol/L compared with control shRNA, with no difference observed at 15 μmol/L. However, these changes were not statistically significant (two-way ANOVA, *P* > 0.05 for all comparisons between *NOTCH3* shRNAs and control). These results indicate that NOTCH3 is not a major target of auranofin in SKOV3 cells. In contrast, *NOTCH3* knockdown with either shRNA in OVCAR3 cells conferred significant resistance to auranofin across the 1.5 to 4 μmol/L concentration range compared with respective controls (Fig. 2C and D; *P* < 0.01). These results confirm that intact NOTCH3 expression is required for auranofin’s cytotoxic effect in OVCAR3 cells.

### Auranofin disrupts RBPJ-Notch–dependent promoter interactions and synergizes with cisplatin in OVCAR3 cells

To confirm that auranofin disrupts RBPJ–DNA interactions, we examined the binding of RBPJ to the promoters of two well-characterized Notch target genes, *HES1* and *HES4*, in OVCAR3 cells using anti-RBPJ ChIP (Fig. 2E). The *CDKN1A* (p21) promoter was used as a negative control. Given that other signaling pathways regulate the expression of *HES1* and *HES4*, a decrease in RBPJ occupancy at NOTCH-dependent promoters is a better surrogate for NOTCH signaling inhibition than a decrease in RNA levels (36). As shown in Fig. 2E, RBPJ was enriched at the *HES1* and *HES4* promoters but not at the p21 promoter (compare dark green with gray bars) in untreated OVCAR3 cells. Treatment with auranofin reduced RBPJ occupancy at the *HES1* and *HES4* promoters in a dose-dependent manner, supporting the hypothesis that Notch signaling is active in OVCAR3 cells and that auranofin inhibits Notch signaling by disrupting RBPJ–DNA interactions.

To determine whether Notch inhibition by auranofin can enhance cisplatin efficacy, as seen with GSIs (14), we treated OVCAR3 cells with cisplatin and auranofin alone or in combination. As shown in Fig. 2F, treatment with 4.7 μmol/L cisplatin or 1.5 μmol/L auranofin alone significantly reduced viability compared with the vehicle. Combined treatment reduced viability more than monotreatment, consistent with synergy. We then applied the Chou–Talalay median-effect method to calculate CIs for cisplatin and auranofin in OVCAR3 (33). Using a 5 × 5 dose matrix (cisplatin: 4–20 μmol/L; auranofin: 1.2–6 μmol/L) and measuring viability at 72 hours, we plotted CI values against the fraction affected. As shown in Fig. 2G, CI values below 1 confirm that cisplatin and auranofin act synergistically to reduce OVCAR3 viability.

### Auranofin treatment increases the efficacy of cisplatin in a CDX mouse model

**Figure 3.**
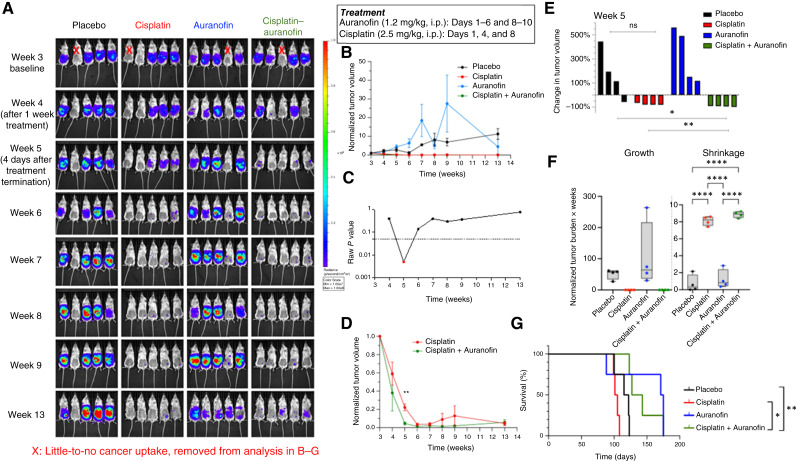
Auranofin increases the overall survival of OVCAR3 CDX mice. **A,***In vivo* imaging system spectrum *in vivo* images of tumor burden. Mice were treated with placebo, cisplatin, auranofin, or both drugs, following the regimen shown. Mice marked with a red “X” in week 3 had little-to-no cancer cell uptake and were removed from data analysis in **B–G**. **B,** Tumor growth over time shown as mean normalized bioluminescence ± SEM for each treatment group (*n* = 4 mice per group at baseline). **C,** Raw *P* values plotted over time comparing cisplatin monotherapy with auranofin–cisplatin combination treatment. Statistical significance (red dot) was determined using multiple unpaired *t* tests. **D,** Magnified view of **B**, focusing on the cisplatin monotherapy and the combination treatment group (scaled between 0 and 1). **E,** Tumor burden for individual mice at week 5. Unpaired *t* tests were used for statistical comparison. **F,** AUC analysis of normalized tumor burden over time for each treatment group. Both tumor growth and shrinkage phases were quantified. Statistical comparisons were performed using one-way ANOVA with Holm–Šídák multiple comparisons. **G,** Kaplan–Meier survival analysis of each treatment group using humane endpoints. Statistical significance was determined by unpaired *t* tests. *, *P* < 0.05; **, *P* < 0.01; ****, *P* < 0.0001.

Next, we used an OVCAR3 xenograft model to examine the effect of auranofin or cisplatin monotreatment and auranofin–cisplatin cotreatment on tumor burden and overall survival (Fig. 3). OVCAR3 cells stably expressing red-shifted firefly luciferase were injected intraperitoneally into NSG female mice (RRID:IMSR JAX:005557). Drug treatment began 3 weeks after engraftment when tumors were well established as detected by bioluminescence imaging. The baseline tumor burden before treatment was quantified for each animal. For our initial OVCAR3 study, a low auranofin dose of 1.2 mg/kg was administered six times a week, and a cisplatin dose of 2.5 mg/kg was administered twice a week (14). Treatment was terminated on day 10 because of cisplatin-related morbidity in cisplatin-sensitive NSG mice. In total, the mice received nine auranofin treatments and three cisplatin treatments (Fig. 3A). Tumor burden was monitored weekly until the animals reached the humane endpoints or death. We first plotted normalized tumor volume as a function of time (Fig. 3B). To determine whether auranofin enhances cisplatin efficacy, we performed Student *t* tests between the cisplatin and combination groups at each time point (Fig. 3C). At week 5 (4 days after treatment ended), cisplatin monotherapy reduced normalized tumor volume to approximately 20% of baseline (Fig. 3D). Strikingly, the cisplatin–auranofin combination drove tumor volume down to ∼5% (*P* < 0.01 vs. cisplatin alone; Fig. 3D). Individual animal responses are shown in Fig. 3E: cisplatin alone produced a nonsignificant reduction compared with placebo (*P* = 0.053), whereas auranofin monotherapy had no significant effect when compared with placebo at this time point. Strikingly, auranofin–cisplatin cotreatment had a statistically significant difference compared with both placebo and cisplatin monotreatment. Moreover, auranofin monotherapy produced a durable response: three of four mice treated with auranofin alone maintained reduced tumor volume through week 13 (Fig. 3A; Supplementary Fig. S2).

Next, we quantified overall changes in tumor burden by calculating the AUC separately for tumor growth and tumor shrinkage (Fig. 3F). Data are presented as a min-to-max box and whisker plot with all individual values. Although no significant differences were observed among the four groups for growth AUC, the combination treatment yielded significantly greater shrinkage AUC than auranofin monotherapy or placebo.

Finally, we analyzed overall survival, defined as time to humane endpoint or death (Fig. 3G). Cisplatin alone did not extend survival in NSG mice, likely reflecting their known sensitivity to cisplatin (Fig. 3G). In contrast, mice receiving the cisplatin–auranofin combination survived significantly longer than those in the placebo or cisplatin groups (Fig. 3G). These results indicate that auranofin not only enhances cisplatin’s antitumor activity but also alleviates its toxicity in this model.

### High-dose auranofin enhances low-dose cisplatin efficacy in OVCAR3 xenografts

**Figure 4.**
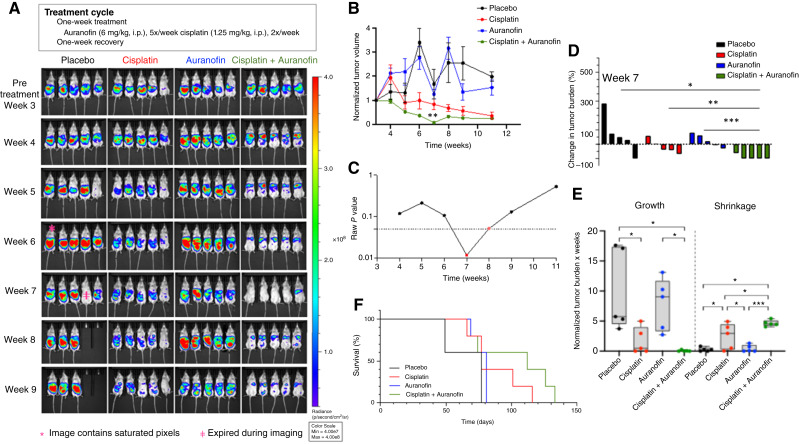
Auranofin synergizes with cisplatin to reduce tumor burden in an OVCAR3 CDX mouse model. **A,** Weekly i*n vivo* imaging system spectrum *in vivo* bioluminescent images showing tumor burden in OVCAR3 CDX mice treated with placebo, cisplatin, auranofin, or both drugs, according to the regimen shown. **B,** Tumor growth over time shown as mean normalized bioluminescence ± SEM for each treatment group (*n* = 5 mice per group at baseline). **C,** Raw *P* values plotted over time comparing cisplatin monotherapy with auranofin–cisplatin combination treatment. Statistical significance (red dots) was determined using multiple unpaired *t* tests. **D,** Tumor burden of individual mice at the end of two treatment cycles (week 7). Unpaired *t* tests were used for statistical comparison. *, *P* < 0.05; **, *P* < 0.01; ***, *P* < 0.001. **E,** AUC analysis of normalized tumor burden over time (weeks 3–13) shown as box and whisker plots. Both tumor growth and shrinkage phases were quantified. Statistical comparisons were performed using one-way ANOVA with Holm–Šídák multiple comparisons. **F,** Kaplan–Meier survival curves. Statistical significance was determined with unpaired *t* tests. *, *P* < 0.05; ***, *P* < 0.001.

Next, we aimed to determine whether a higher auranofin dose could further enhance the efficacy of cisplatin while simultaneously reducing cisplatin exposure to minimize toxic side effects in the OVCAR3 xenograft model. To achieve this goal, we devised a biweekly regimen in which the mice were treated every other week with 6 mg/kg auranofin and 1.25 mg/kg cisplatin (Fig. 4A).

Four groups of five mice each were engrafted with OVCAR3 cells stably expressing red-shifted firefly luciferase, and the tumor burden was quantified weekly by bioluminescent imaging (Fig. 4A). Biweekly cisplatin treatment was well tolerated by the mice. As shown in Fig. 4B, combination-treated mice exhibited a more consistent reduction in tumor burden during treatment than those receiving cisplatin alone. Focused *t* tests at weeks 7 (*P* = 0.012) and 8 (*P* ≈ 0.051) confirmed that the combination therapy outperformed cisplatin monotherapy (Fig. 4C). Data for weeks 5 and 9, which did not reach statistical significance, are shown in Supplementary Fig. S3. After two treatment cycles (week 7), the combination group’s tumor burden was significantly lower than that of the placebo, cisplatin, or auranofin groups (Fig. 4D).

To assess overall tumor dynamics, we calculated individual mouse AUCs from weeks 3 through 11, separating growth AUC from shrinkage AUC (Fig. 4E). One-way ANOVA revealed significant effects on both growth and shrinkage AUC (*P* < 0.01). Holm–Šídák multiple comparisons showed that cisplatin monotherapy reduced growth AUC versus placebo (*P* < 0.05) and that the combination of cisplatin plus auranofin further reduced growth AUC compared with both auranofin alone (*P* < 0.05) and placebo (*P* < 0.05). For shrinkage AUC, the combination treatment increased shrinkage relative to cisplatin alone (*P* < 0.05) and to placebo (*P* < 0.05). Notably, variance in growth AUC was lowest in the combination group (F-test, *P* < 0.001), indicating more uniform tumor control. Finally, overall survival (Fig. 4F) showed a trend toward improvement in both the cisplatin and combination groups. Altogether, these data indicate that auranofin enhances cisplatin efficacy and leads to more consistent tumor reduction.

### NOTCH3 signaling is active across HGSOC organoid models but does not correlate with platinum sensitivity

**Figure 5.**
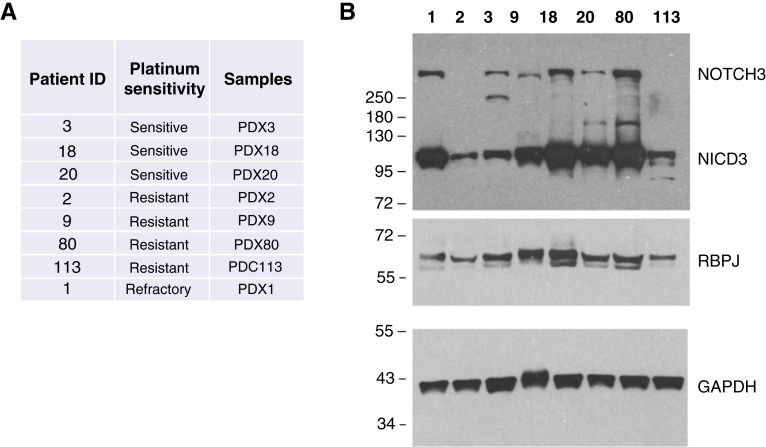
Patient-derived tumor models used in this study. **A,** Clinical platinum sensitivity of the patients with HGSOC from whom the organoid models were derived. **B,** Western blot analysis of total cell lysates showing NOTCH3 and RBPJ protein levels in eight patient-derived models. GAPDH was used as the loading control.

We used patient-derived ovarian cancer organoids from eight patients with HGSOC to assess interpatient variability in auranofin’s enhancement of cisplatin efficacy (Fig. 5). Cancer organoid models were derived from a diverse cohort of patients, including platinum-sensitive patients (patients 3, 18, and 20), platinum-resistant cases (recurrence within 6 months of treatment, patients 2, 9, 80, and 113), and one platinum-refractory case (no response to first-line therapy, patient 1). These models are shown in Fig. 5A. NOTCH3 and RBPJ protein levels in PDX and ascites samples were determined by Western blot analysis (Fig. 5B). RBPJ and NICD3 were detected in all eight samples, consistent with the notion that NOTCH3 signaling is broadly active in this disease context. Among the models, PDX2, PDX3, and PDX113 showed the lowest relative levels of NICD3 (Table 1). All other models had relatively high levels of NICD3 protein expression. A direct correlation between NICD3 abundance and platinum response was not observed.

**Table 1.** NICD3 levels and auranofin IC_50_ in PDOs.

Model	NICD3/GAPDH	IC_50__AuR (μmol/L)^a^
High NICD3 (≥1.5)		
PDO1	1.9	2.7
PDO18	1.7	2.9
PDO20	1.7	2.7
PDO80	1.6	1.8
Mid NICD3 (1.0–1.5)		
PDXO9	1.0	0.8
Low NICD3 (<1.0)		
PDO2	0.7	33.0
PDO113	0.4	13.0
PDXO3^b^	0.5	0.4

a These values were calculated from the data shown in Figs. 6 and 7 using a constrained three-parameter logistic fit.

b PDXO3 shows exceptional auranofin (AuR) sensitivity despite low NICD3.

### Auranofin restores platinum sensitivity in cancer organoids derived directly from two platinum-resistant patients

We evaluated auranofin’s modulation of cisplatin cytotoxicity in organoids from five platinum-resistant or platinum-refractory patients (Fig. 6; Supplementary Fig. S4). The upper panels of Fig. 6 (boxed inset, top right) present dose–response curves for varying cisplatin concentrations at fixed auranofin levels, and the lower panels (boxed inset, bottom right) show the reciprocal curves for varying auranofin concentrations at fixed cisplatin levels. In PDO113 and PDO2, 1 μmol/L or 4 μmol/L cisplatin alone had no effect on viability over 3 days, confirming resistance (Fig. 6A and B). Cotreatment with auranofin (1, 2.5, or 7.5 μmol/L) markedly enhanced cisplatin efficacy, as evidenced by steeper dose–response slopes and greater viability reduction; conversely, cisplatin (1 or 4 μmol/L) potentiated auranofin activity. Statistical analyses of replicate experiments are shown in Supplementary Fig. S4A and S4B (*P* values indicated). Notably, PDO2 and its paired PDX-derived organoid model PDXO2 exhibited concordant responses (Fig. 6B; Supplementary Figs. S4B, S5A, S5B, and S6A), demonstrating that organoids derived directly from patient tumors or xenografts behave similarly. By contrast, auranofin did not restore platinum sensitivity in PDO1, PDO80, or PDXO9 (Fig. 6C–E; Supplementary Fig. S4C–S4E) nor in the PDXO1 model (Supplementary Figs. S5C, S5D, and S6B). These outcomes, which mirror findings with other Notch pathway inhibitors (14, 37), likely reflect the multiple mechanisms of drug resistance (e.g., increased efflux).

**Figure 6.**
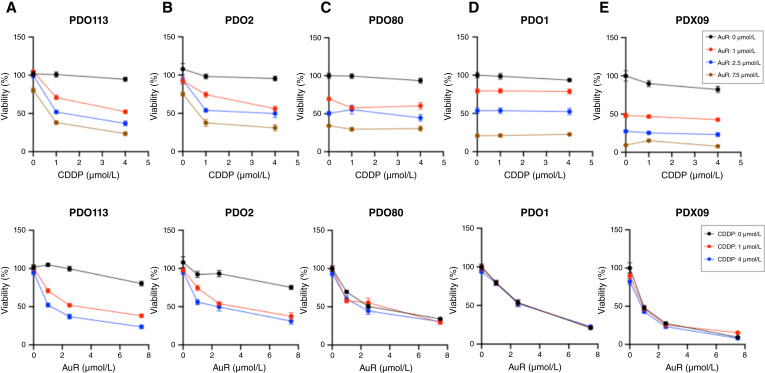
Auranofin effects on platinum sensitivity of cancer organoids. Viability assays were performed on five organoid models. **A,** PDO113, (**B**) PDO2, and (**C**) PDO80 were derived directly from platinum-resistant patients. **D,** PDO1 was derived directly from a platinum-refractory patient. **E,** PDXO9 was developed from a patient-derived xenograft model. In each panel, the top graph shows cisplatin dose–response curves measured in the presence of fixed concentrations of auranofin (see inset), whereas the bottom graph shows auranofin dose–response curves measured in the presence of fixed concentrations of cisplatin (see inset). All assays were performed 72 hours after drug additions. Data are plotted as means ± SEM (*n* ≥ 5). Statistical comparisons are provided in Supplementary Fig. S4. AuR, auranofin; CDDP, cisplatin.

To test whether auranofin inhibits NOTCH signaling, we performed anti-RBPJ ChIP-qPCR in PDXO1. As shown in Supplementary Fig. S5E, auranofin dose-dependently disrupted RBPJ binding at the Notch-dependent *HES1* and *HES4* promoters, but not at the negative control region (p21).

### Auranofin increases the efficacy of cisplatin in killing cancer organoids derived from three platinum-sensitive patients

**Figure 7.**
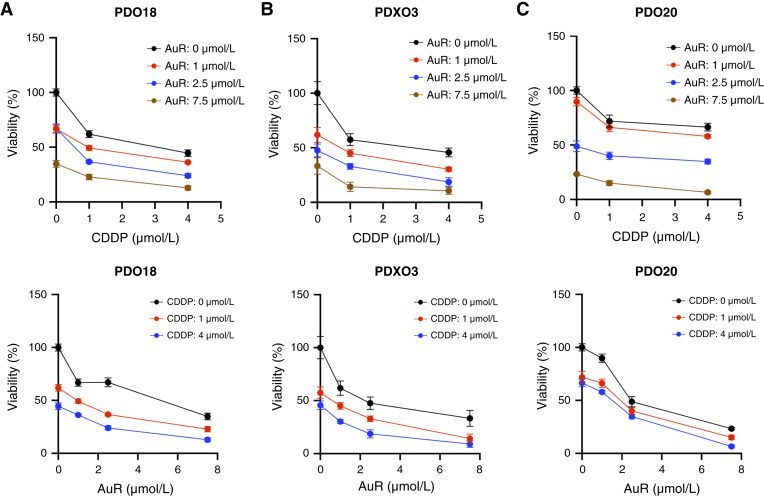
Auranofin enhances the efficacy of cisplatin in cancer organoids derived from platinum-sensitive patients. Viability assays were performed as described in Fig. 6. Cancer organoids were derived from patients clinically classified as platinum sensitive. **A,** PDO18; (**B**) PDXO3, developed from a patient-derived xenograft model; and (**C**) PDO20. Data are presented as means ± SEM (*n* ≥ 5 technical replicates). Statistical analyses are shown in Supplementary Fig. S7. AuR, auranofin; CDDP, cisplatin.

Next, we tested the responses of clinically defined platinum-sensitive samples (PDO18, PDO20, and PDXO3) to auranofin and cisplatin cotreatment (Fig. 7; Supplementary Fig. S7). All three organoid models were sensitive to cisplatin monotherapy, which was consistent with their clinical response to platinum-based therapy. All three organoid models demonstrated a response to cisplatin and auranofin combination therapy, but to varying degrees. For PDO18, cisplatin at 1 and 4 μmol/L decreased organoid viability in a dose-dependent manner, and auranofin significantly enhanced cisplatin efficacy at all concentrations tested (Fig. 7A; Supplementary Fig. S7A). Similar results were obtained for PDXO18 (Supplementary Fig. S6C). Synergy was also observed with PDXO3, with auranofin concentrations of 1 and 2.5 μmol/L enhancing the efficacy of 4 μmol/L cisplatin (Fig. 7B; Supplementary Fig. S7B). For PDO20, auranofin consistently enhanced the efficacy of cisplatin at 4 μmol/L (Fig. 7C); however, the specific auranofin concentration at which synergy occurred varied between biological replicates (*n* = 2; Supplementary Fig. S7C and S7D). Together, the data from these organoid models suggest that auranofin could be used in combination with cisplatin to treat platinum-sensitive tumors.

### High NICD3 expression in organoids negatively correlates with auranofin IC_50_

Although auranofin did not enhance cisplatin efficacy in some organoids expressing NICD3, it demonstrated dose-dependent cytotoxicity on its own. We therefore quantified NICD3 levels in patient-derived samples (Fig. 5B) and determined auranofin IC_50_ values from our drug–response assays (Figs. 6 and 7); results are summarized in Table 1. PDOs with high NICD3 expression (≥1.5) consistently displayed low IC_50_ values (1.8–2.9 μmol/L), whereas those with low NICD3 (<1.0) were markedly less sensitive (13–33 μmol/L). A Pearson correlation analysis excluding PDXO3 confirmed a moderate inverse relationship between NICD3 level and auranofin IC_50_ (r = −0.66, one-tailed *P* = 0.055). Notably, PDXO3 exhibited a low IC_50_ (0.4 μmol/L) despite its low NICD3 expression.

## Discussion

In this study, we assessed the utility of auranofin for the treatment of ovarian cancer using established monolayer cell lines, CDX mouse models, and patient-derived organoids. Using shRNA, we demonstrate that Notch3 is a major auranofin target in high-NICD3 OVCAR3 but not in low-NICD3 SKOV3 cells. Using anti-RBPJ ChIP-qPCR, we demonstrate that in both ovarian cancer cells and organoids, auranofin disrupts the interaction of RBPJ with the promoters of Notch target genes in a dose-dependent manner (Fig. 2E; Supplementary Fig. S4E), as previously observed in F9 and 293T cells as well as *in vitro* reconstituted systems (30). Additionally, we show that auranofin enhances cisplatin efficacy, a standard chemotherapeutic for ovarian cancer, in NOTCH-dependent OVCAR3 cells (Fig. 2F and G; refs. 12, 14). *In vivo*, auranofin cotreatment both reduced cisplatin toxicity and extended survival in OVCAR3 CDX models; notably, this combination produced significantly greater tumor regression than either drug alone (Figs. 3 and 4). In patient-derived organoids, auranofin enhanced cisplatin sensitivity and, as a single agent, produced dose-dependent killing in NOTCH-activated models (Figs. 5–7; Table 1). One low-NICD3 organoid (PDXO3) remained sensitive (IC_50_ = 0.4 μmol/L), potentially due to enhanced uptake or proapoptotic epigenetic/mutational features. Together, these findings establish Notch3 activity as a key determinant of auranofin potency and support its development, alone or in combination with cisplatin, for personalized ovarian cancer therapy.

Although our experimental work evaluated cisplatin, carboplatin with paclitaxel is the current first-line standard of care for epithelial ovarian cancer. Cisplatin and carboplatin form similar DNA adducts and share many resistance determinants, so cross-resistance is common. Given that auranofin enhanced cisplatin efficacy in NOTCH-dependent ovarian cancer models and restored platinum response in a subset of platinum-resistant organoids, we hypothesize that auranofin could also resensitize carboplatin-resistant disease. Future testing of auranofin with carboplatin in patient-derived organoids and *in vivo* models will define the breadth of activity across platinum contexts.

Auranofin was approved by the FDA in 1985 to treat rheumatoid arthritis, as it elicits an anti-inflammatory response, although the underlying mechanism that leads to this response is still unclear. Intriguingly, aberrant NOTCH signaling has been linked to autoimmune inflammatory diseases (38, 39), supporting our findings (30). Auranofin has additional targets, including thioredoxin reductase 1, a redox regulator that is the most well-studied (40, 41). Consequently, auranofin was tested in a clinical trial (NCT01419691) for the treatment of chronic lymphocytic leukemia (CLL) based on the premise that CLL cells exhibit higher basal reactive oxygen species levels (42). Auranofin treatment was predicted to elevate cellular reactive oxygen species levels and preferentially reduce the viability of CLL cells compared with normal lymphocytes. In a second early phase I clinical study (NCT01747798), auranofin was tested for the treatment of ovarian cancer, based on evidence that it inhibits protein kinase C iota, a serine–threonine protein kinase associated with ovarian cancer tumorigenesis (43). Although conceptually promising, follow-up studies of these clinical trials have not yet been reported.

We observed that auranofin (1.2 mg/kg) enhanced cisplatin efficacy in OVCAR3 xenografts, which was most evident 4 days after the end of treatment (Fig. 3A–E, week 5). At this concentration and time, the tumor burden in mice treated with auranofin monotherapy was similar to that in the placebo control. However, auranofin had a delayed effect, reducing tumor burden in three out of four mice by 8 weeks after treatment termination (Fig. 3A; Supplementary Fig. S2, week 13). Increasing the dose of auranofin to 6 mg/kg did not enhance its therapeutic effect. Owing to toxicity, we could not further increase the auranofin concentration. The delayed therapeutic effect of auranofin that we observed may result from the time required for genetic reprogramming and cell-cycle withdrawal. Alternatively, but not mutually exclusively, NOTCH signaling is involved in angiogenesis (19, 44, 45); therefore, auranofin treatment might have lasting effects by restricting the vascular network that supports tumor growth. Further studies using PDX models with varying expression levels of NOTCH3 will provide additional insights. Regardless, our observation that a low dose of auranofin (1.2 mg/kg) rescued cisplatin-induced cell toxicity (Fig. 3E) is of great interest and worthy of further investigation.

Although PDO18 and PDO20 both exhibit robust NICD3 expression, they remain platinum-sensitive, indicating that NOTCH3 activation alone is insufficient to confer cisplatin resistance. Across our PDO panel, the lack of a strict correlation between NICD3 levels and platinum phenotype suggests that resistance is multifactorial even in NOTCH-active tumors. Nonetheless, aberrant NOTCH signaling is a major driver of resistance to platinum-based compounds (12, 14), and our data show that auranofin restores cisplatin sensitivity in a subset of platinum-resistant organoids and enhances cisplatin’s effect on tumor burden *in vivo*, similar to results reported with GSIs (14, 26). Because auranofin is FDA-approved with a well-characterized pharmacology, it may offer an option directed at the NOTCH pathway with improved tolerability relative to current inhibitors. Consistent with the clinical activity reported for the anti-DLL4 antibody demcizumab in heavily pretreated, platinum-resistant ovarian cancer (clinical benefit rate 40%, NCT01952249; refs. 37, 46), we observed a 40% response rate in our platinum-resistant PDOs (two of five models; Fig. 6; Supplementary Fig. S4), supporting further evaluation of auranofin in platinum-resistant disease.

In our study, baseline NICD3 abundance did not reliably predict which PDOs would benefit from auranofin plus platinum cotherapy, suggesting that other factors shape the response. To address this, we envision a PDO-guided approach to patient selection, using short-turnaround assays on ascites or tumor biopsies to identify candidates for the combination. These assays capture the diversity seen between patients and have been shown to align with clinical outcomes.

## Supplementary Material

Figure S1 Supplementary Figure S1 shows crystal violet–stained SKOV3 (A) and OVCAR3 (B) cells after *NOTCH3* knockdown (shRNA_A, shRNA_C), following 7-day puromycin selection, with lower panels providing higher-magnification views of representative colonies.

Figure S2 Supplementary Figure S2 displays individual bioluminescent tumor burden measurements in OVCAR3 cell–derived xenograft mice at week 13 via IVIS Spectrum; significance was assessed by multiple unpaired t-tests (ns, p > 0.05).

Figure S3 Supplementary Figure S3 quantifies in vivo bioluminescent tumor burden in individual OVCAR3 cell–derived xenograft mice at weeks 5 and 9 using IVIS Spectrum; unpaired t-tests indicated no significant differences at these time points (*P < 0.05; **P < 0.01).

Figure S4 Supplementary Figure S4 shows representative viability assays of platinum-resistant patient-derived cancer organoids treated with cisplatin (CDDP), auranofin (AuR), or mock for 72 hours; concentrations (µM) are indicated. Data are mean ± SD (n > 5), with one-way ANOVA and Holm–Sidak multiple comparisons highlighting only significant effects of AuR monotherapy (****P < 0.0001; ***P < 0.001; **P < 0.01; *P < 0.05).

Figure S5 Supplementary Figure S5 presents responses of PDX-derived cancer organoids to cisplatin, auranofin, or their combination versus mock treatment: panels A–B show PDXO2 and panels C–D show PDXO1 (mean ± SEM, n > 5). Panel E displays anti-RBPJ ChIP-qPCR in PDXO1 organoids treated with DMSO or auranofin (0.8 µM, 2.5 µM) for 24 hours (mean ± SEM, n = 3 technical replicates), evaluating RBPJ enrichment at HES1 and HES4 promoters versus beads-only control by paired two-tailed t-tests (**P < 0.01; *P < 0.05; ns, P > 0.05).

Figure S6 Supplementary Figure S6 shows viability responses of PDX-derived cancer organoids (PDXO2, PDXO1, PDXO18) to cisplatin, auranofin, or their combination versus mock treatment; data are presented as mean ± SD (n > 5), with one-way ANOVA and Holm–Sidak multiple comparisons indicating only significant synergistic interactions (****P < 0.0001; ***P < 0.001; **P < 0.01; *P < 0.05; ns, P > 0.05).

Figure S7 Supplementary Figure S7 presents viability assays for platinum-sensitive patient-derived cancer organoids (PDO18, PDXO3, PDO20, replicate PDO20) treated with auranofin ± cisplatin, with data as mean ± SD (n > 5) and one-way ANOVA + Holm–Sidak for synergy (****P < 0.0001; ***P < 0.001; **P < 0.01; *P < 0.05).

Table S1 Table S1 lists the primers used in ChIP-qPCR

## Data Availability

The data generated in this study are available in the article and supplemental files or from the corresponding authors upon request.
