# Supplementary figures and images for: Auranofin Synergizes with Cisplatin in Reducing Tumor Burden of NOTCH-Dependent Ovarian Cancer
Source: Cancer Res Commun. 2025 Oct 10;5(10):1796–808. doi: 10.1158/2767-9764.CRC-25-0190 (PMC12512110; doi:10.1158/2767-9764.CRC-25-0190)

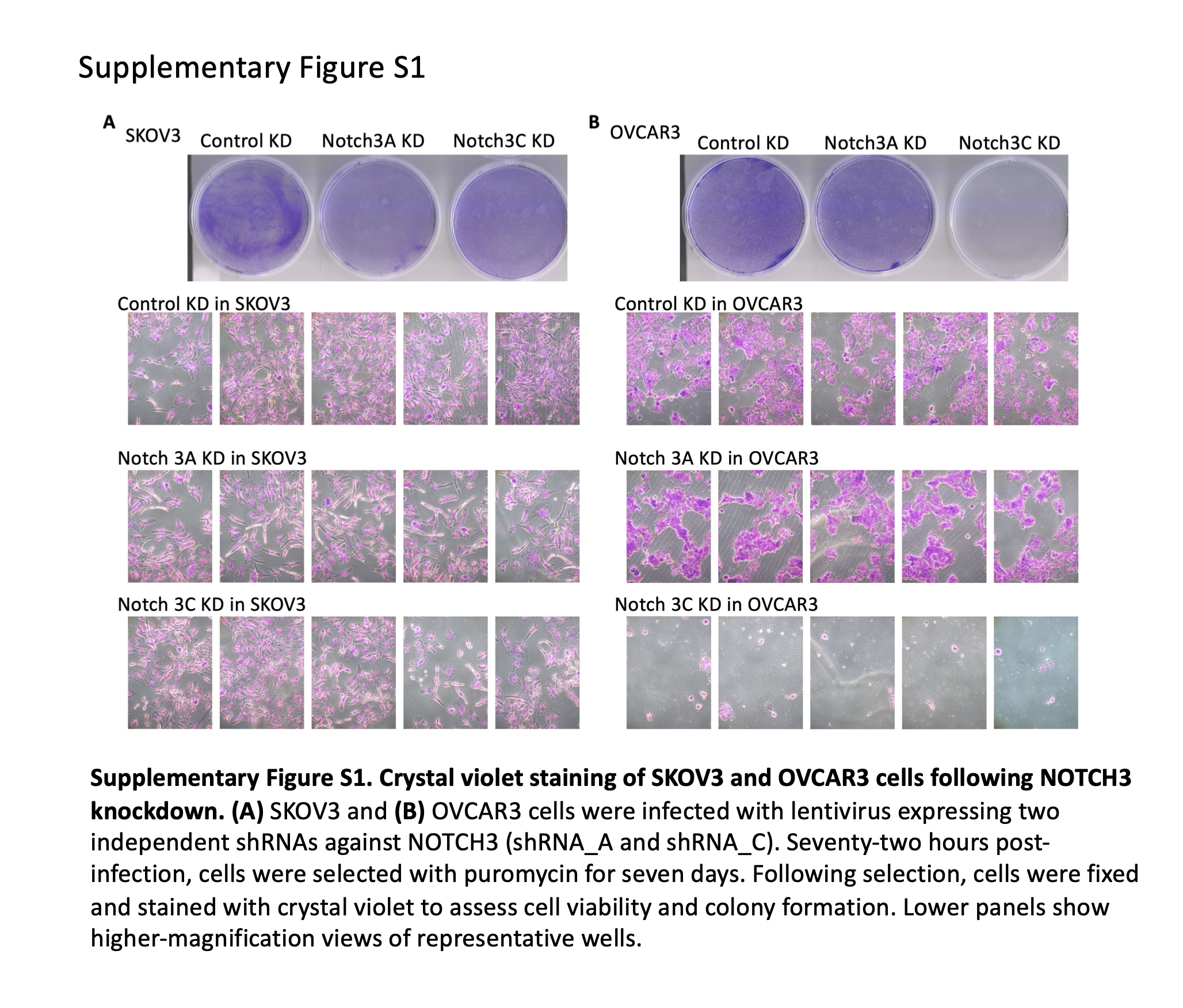

Supplement: Figure S1 — Supplementary Figure S1 shows crystal violet–stained SKOV3 (A) and OVCAR3 (B) cells after NOTCH3 knockdown (shRNA_A, shRNA_C), following 7-day puromycin selection, with lower panels providing higher-magnification views of representative colonies. [file crc-25-0190_figure_s1_suppsf1.png]

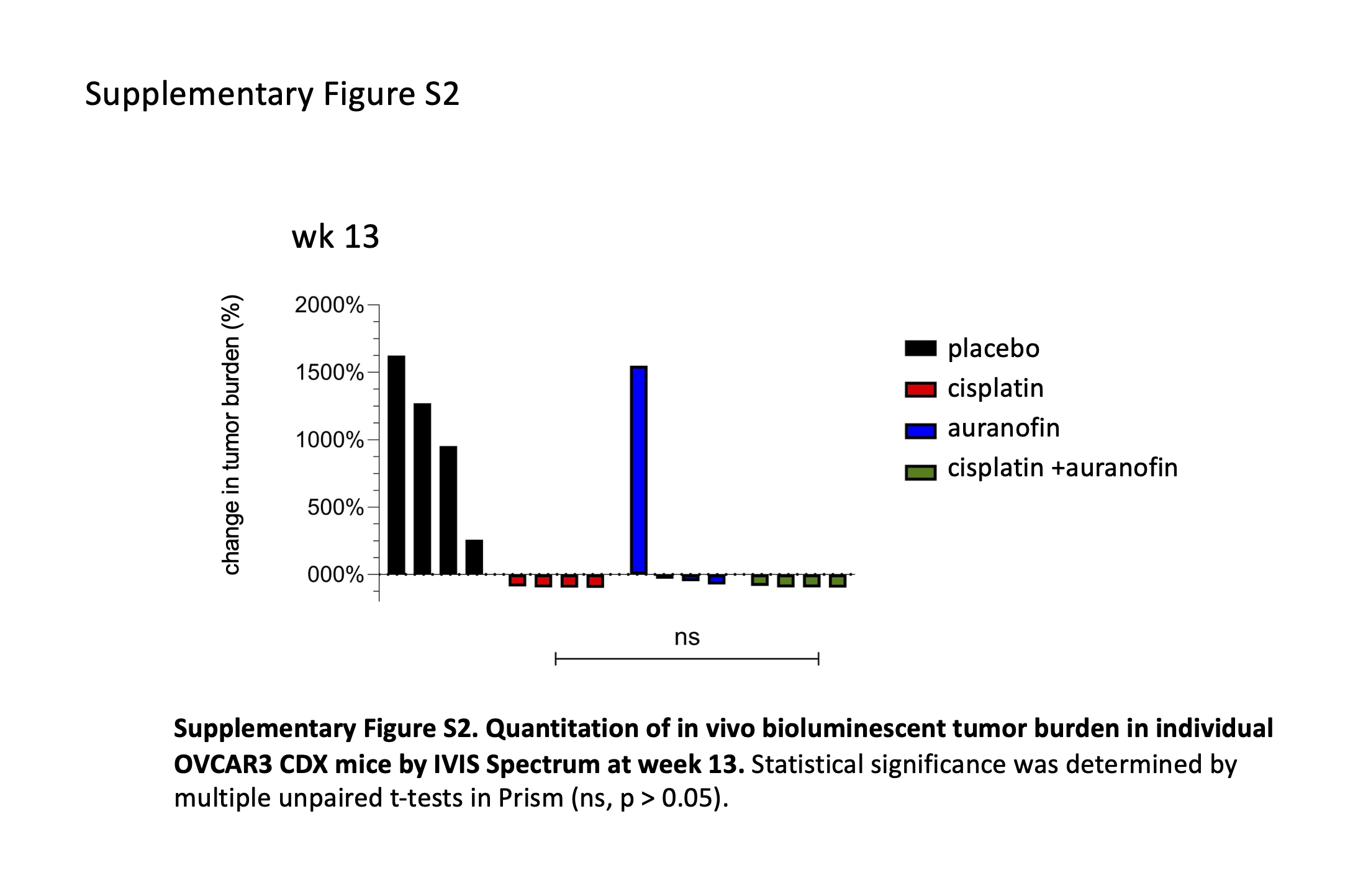

Supplement: Figure S2 — Supplementary Figure S2 displays individual bioluminescent tumor burden measurements in OVCAR3 cell–derived xenograft mice at week 13 via IVIS Spectrum; significance was assessed by multiple unpaired t-tests (ns, p > 0.05). [file crc-25-0190_figure_s2_suppsf2.png]

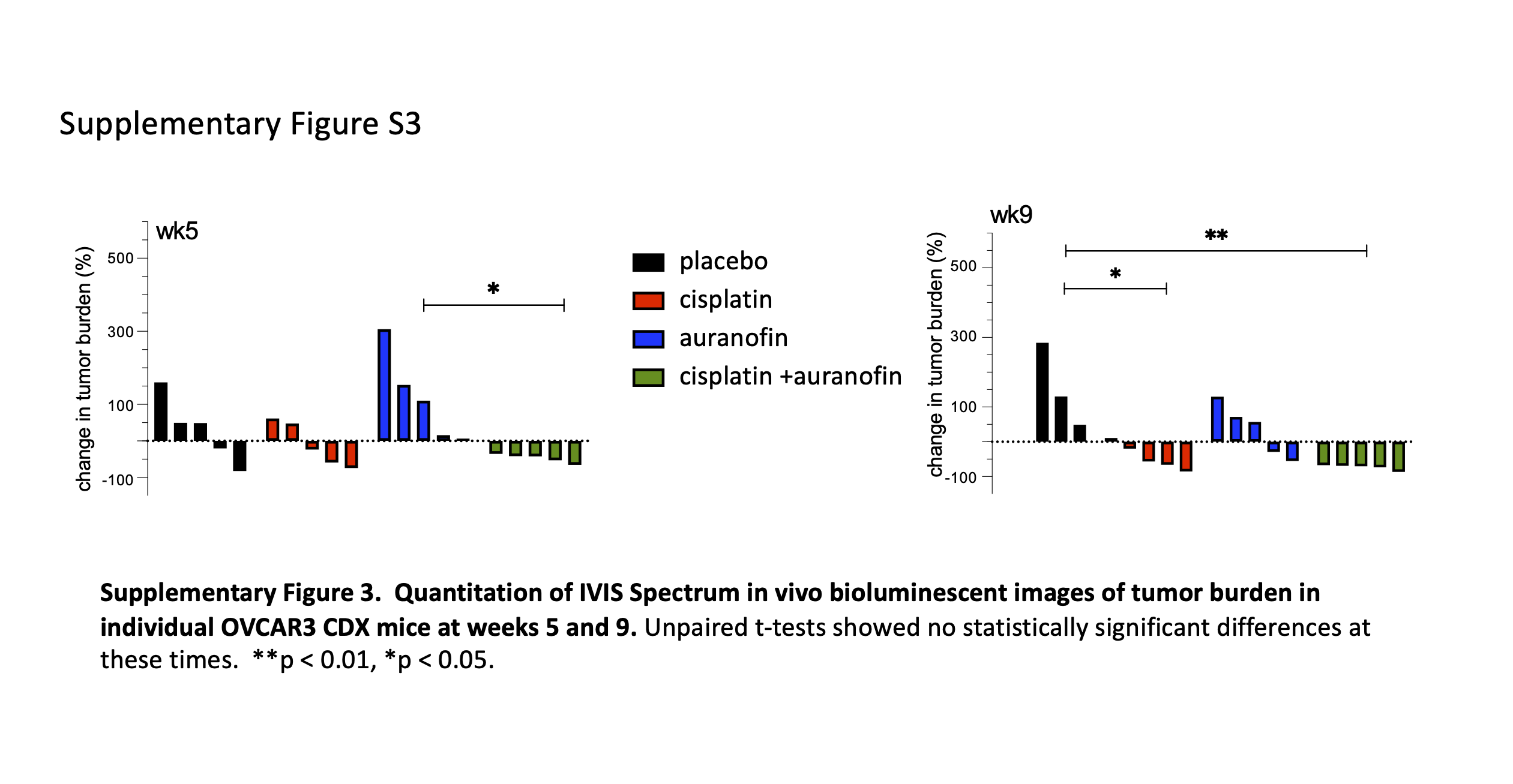

Supplement: Figure S3 — Supplementary Figure S3 quantifies in vivo bioluminescent tumor burden in individual OVCAR3 cell–derived xenograft mice at weeks 5 and 9 using IVIS Spectrum; unpaired t-tests indicated no significant differences at these time points (*P < 0.05; **P < 0.01). [file crc-25-0190_figure_s3_suppsf3.png]

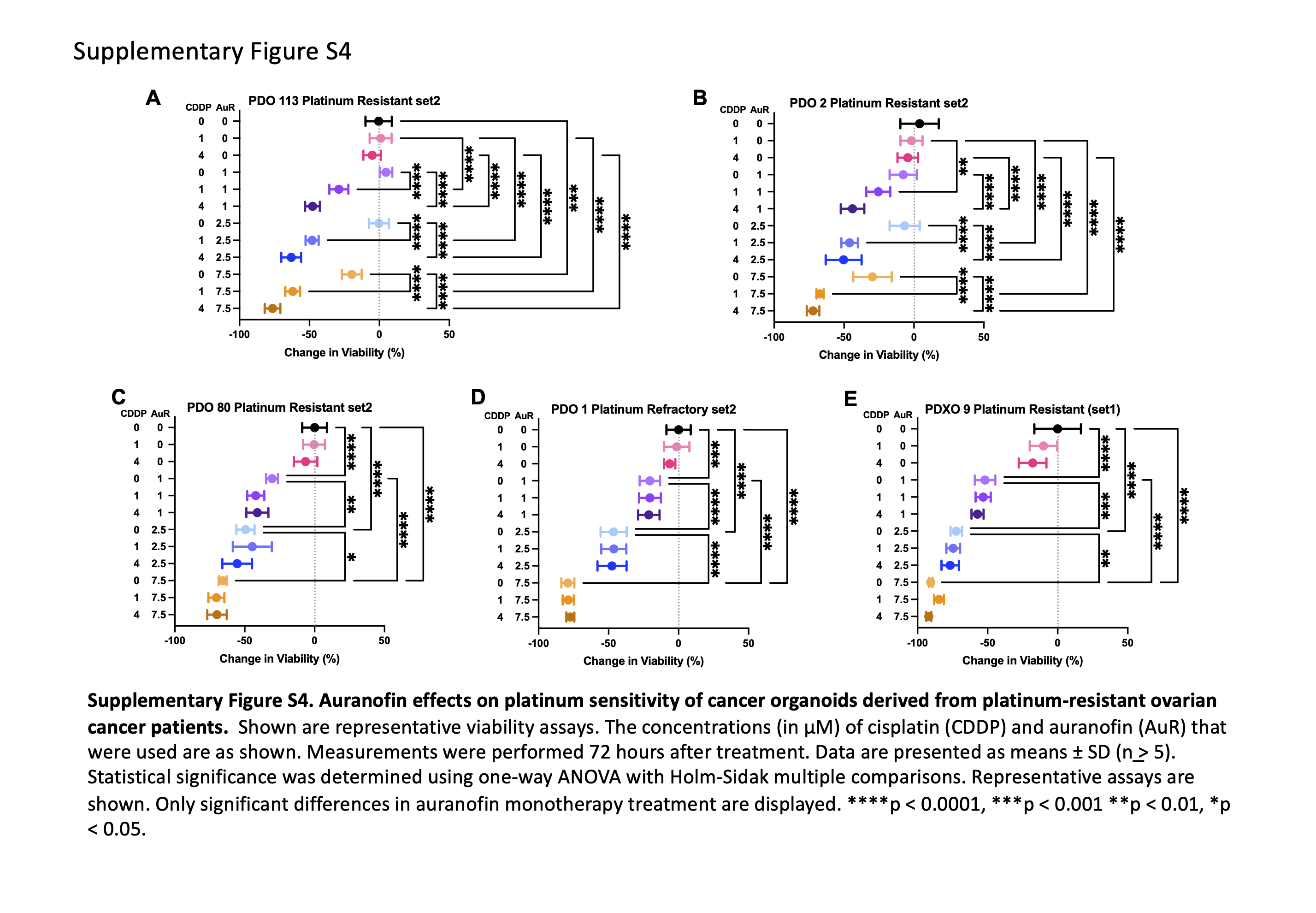

Supplement: Figure S4 — Supplementary Figure S4 shows representative viability assays of platinum-resistant patient-derived cancer organoids treated with cisplatin (CDDP), auranofin (AuR), or mock for 72 hours; concentrations (µM) are indicated. Data are mean ± SD (n > 5), with one-way ANOVA and Holm–Sidak multiple comparisons highlighting only significant effects of AuR monotherapy (****P < 0.0001; ***P < 0.001; **P < 0.01; *P < 0.05). [file crc-25-0190_figure_s4_suppsf4.png]

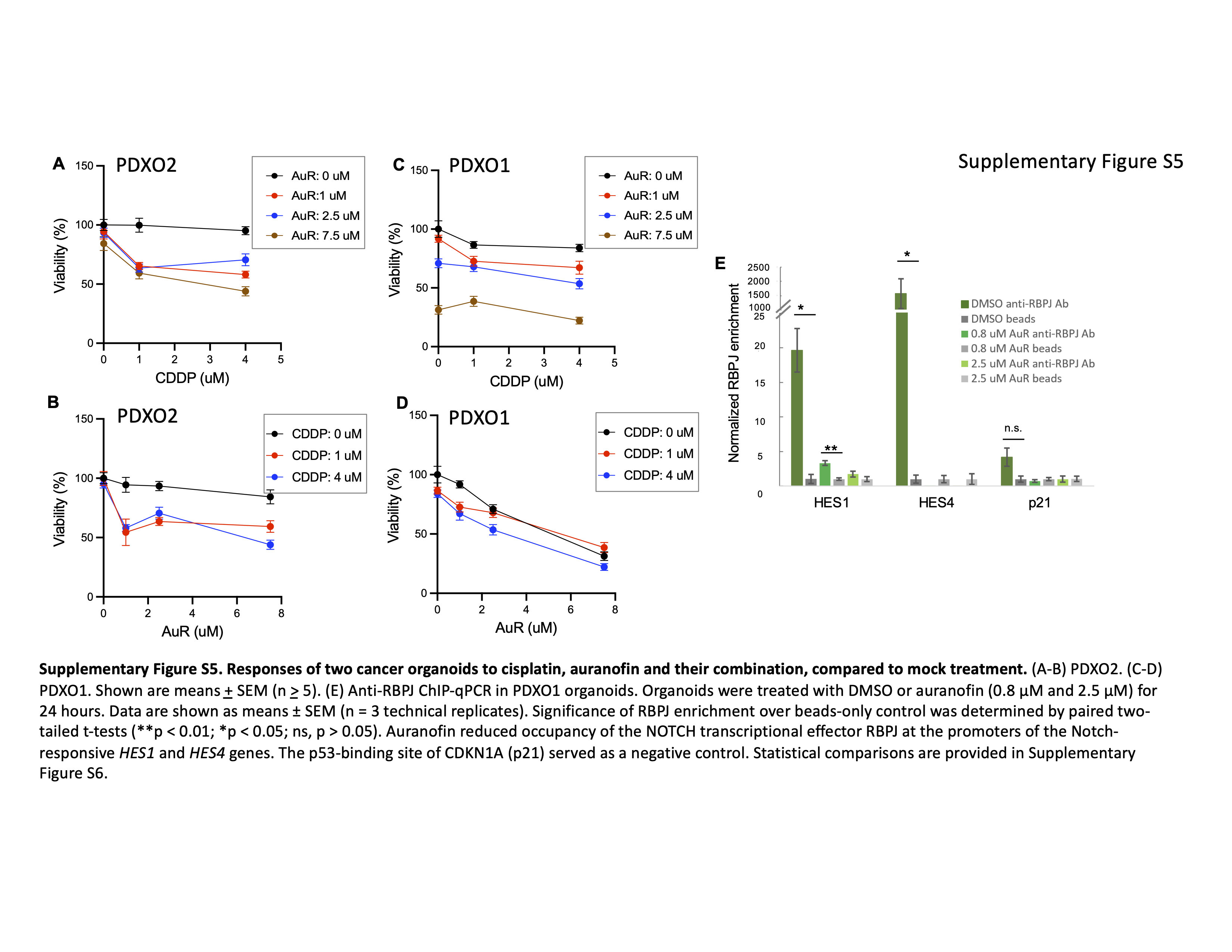

Supplement: Figure S5 — Supplementary Figure S5 presents responses of PDX-derived cancer organoids to cisplatin, auranofin, or their combination versus mock treatment: panels A–B show PDXO2 and panels C–D show PDXO1 (mean ± SEM, n > 5). Panel E displays anti-RBPJ ChIP-qPCR in PDXO1 organoids treated with DMSO or auranofin (0.8 µM, 2.5 µM) for 24 hours (mean ± SEM, n = 3 technical replicates), evaluating RBPJ enrichment at HES1 and HES4 promoters versus beads-only control by paired two-tailed t-tests (**P < 0.01; *P < 0.05; ns, P > 0.05). [file crc-25-0190_figure_s5_suppsf5.png]

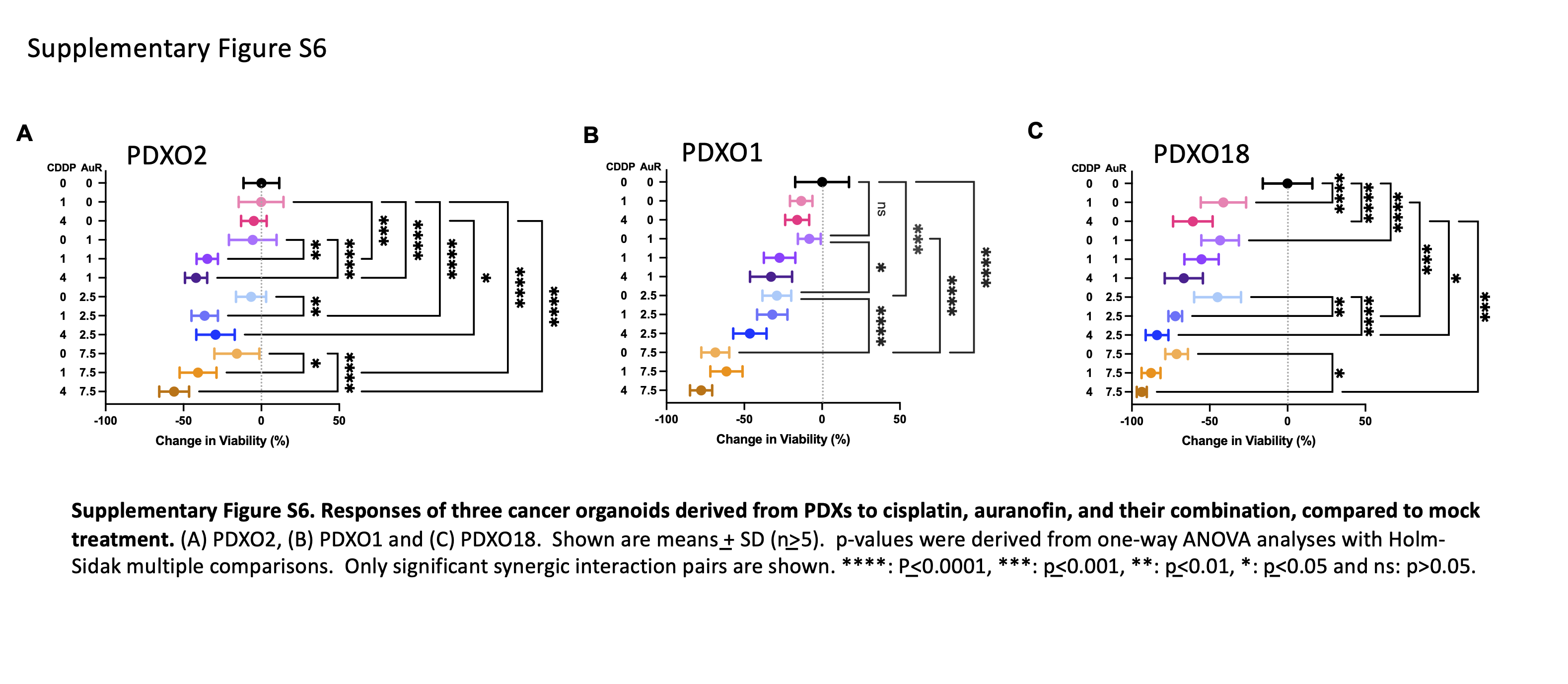

Supplement: Figure S6 — Supplementary Figure S6 shows viability responses of PDX-derived cancer organoids (PDXO2, PDXO1, PDXO18) to cisplatin, auranofin, or their combination versus mock treatment; data are presented as mean ± SD (n > 5), with one-way ANOVA and Holm–Sidak multiple comparisons indicating only significant synergistic interactions (****P < 0.0001; ***P < 0.001; **P < 0.01; *P < 0.05; ns, P > 0.05). [file crc-25-0190_figure_s6_suppsf6.png]

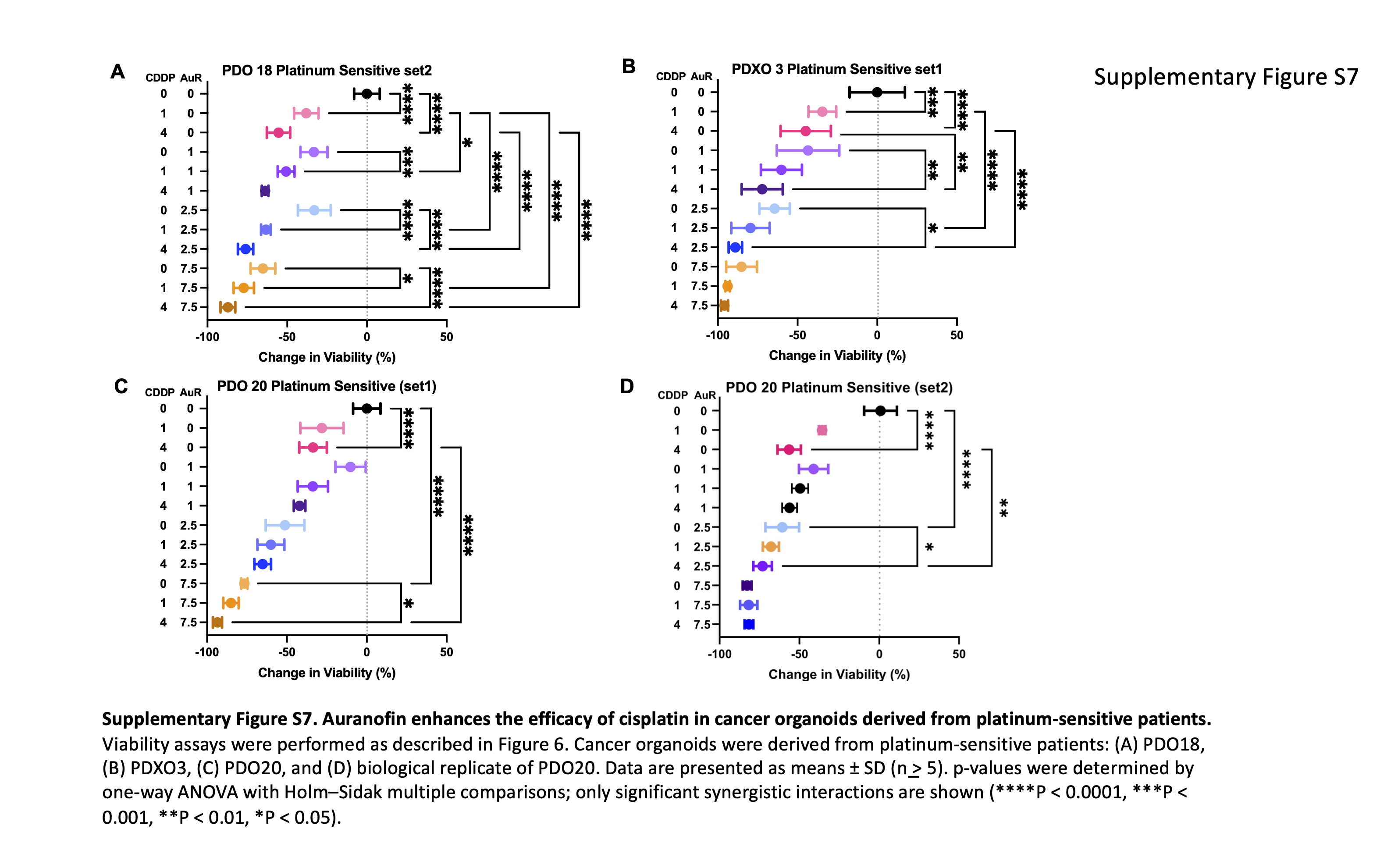

Supplement: Figure S7 — Supplementary Figure S7 presents viability assays for platinum-sensitive patient-derived cancer organoids (PDO18, PDXO3, PDO20, replicate PDO20) treated with auranofin ± cisplatin, with data as mean ± SD (n > 5) and one-way ANOVA + Holm–Sidak for synergy (****P < 0.0001; ***P < 0.001; **P < 0.01; *P < 0.05). [file crc-25-0190_figure_s7_suppsf7.png]
